# Analyzing the patient isocenter positional accuracy with portal imaging acquired at different gantry angles and its dosimetrical impact on the dose delivery for head and neck cancers

**DOI:** 10.1002/pro6.1234

**Published:** 2024-06-28

**Authors:** Bharath Pandu, D Khanna, Mohandass Palanisamy, Saro Jacob, Tatarao Maddipati

**Affiliations:** ^1^ Department of Applied Physics Karunya Institute of Technology and Sciences Coimbatore India; ^2^ Department of Radiotherapy Bangalore Baptist Hospital Bangalore India; ^3^ Department of Radiation Oncology Fortis Hospital Mohali Mohali India; ^4^ Department of Community Medicine Bangalore Baptist Hospital Bangalore India

**Keywords:** electronic portal imaging devices, gantry angles, intensity‐modulated radiotherapy, isocenter shift, portal imaging, positional accuracy

## Abstract

**Objective:**

This study aimed to analyze the effect of different gantry angles on portal imaging in terms of isocenter shifts and their dosimetric impact on dose delivery.

**Methods:**

Thirty patients with head and neck cancers were prospectively selected for this study. The reference anterior to posterior (AP) digitally reconstructed reference radiograph image was obtained at a gantry angle of 0°. The AP portal images were acquired at angles of 0°, 0.5°, 1°, 1.5°, and 2.0°. The average deviation of the isocenter shift with respect to the zero‐gantry angle was calculated. To check the dosimetric effects, the reference fluence was compared with different fluences measured at different isocenter shifts.

**Results:**

The average isocenter shift differences in the lateral direction were 0.7 mm, 1.3 mm, 1.9 mm, and 2.5 mm. The average difference was <±0.1 mm for isocenter shift in the longitudinal direction. The results of the statistical analysis showed that the average isocenter shift and gamma pass rate with respect to the different isocenter position errors were significant in the lateral direction.

**Conclusion:**

The results of this study showed that as the gantry angle increased, the isocenter shifted and the percentage of deviation in the lateral direction increased.

## Graphical abstract

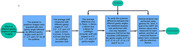

## 1 INTRODUCTION

In modern radiotherapy, various advanced treatment modalities such as intensity‐modulated radiotherapy (IMRT), volumetric modulated arc therapy, stereotactic radiosurgery (SRS), and stereotactic body radiotherapy are used for radiotherapy.^1^ In all these treatment techniques, the treatment is planned with a sharp dose fall off to spare normal tissue. To spare normal tissue, the margin of the planning target volume (PTV) was reduced. Owing to the reduced margin in the PTV, patient positioning must be precise to deliver the planned treatment dose accurately. In the last three decades, to check patient positioning, external beam radiotherapy with high‐energy photon beams has required verification of the isocenter before treating each patient.^2^ Electronic portal imaging devices (EPID) and cone beam computed tomography (CBCT) play important roles in verifying the accuracy of the isocenter of external beam radiotherapy during patient treatment.^3^ The performance of EPID and CBCT is crucial to ensure the accuracy of the treatment position and reproducibility of patient positioning.^4^ The purpose of image verification is to perform treatments with accurate patient positioning.^5^ When the treatment position is accurately verified, the treatment is delivered accurately; the tumor receives the prescribed dose, and normal tissues receive the planned dose.^6^, ^7^ The dose distribution of the IMRT treatment plan is delivered incorrectly if the patient is positioned incorrectly or if the patient moved during treatment.^8^ Several radiotherapy centers do not have the facilities to perform CBCT. These treatment centers use EPID‐based setup verification for IMRT and other advanced treatment modalities.

During daily patient setups, setup and target motion errors can occur.^9^, ^10^ To improve patient positioning during radiotherapy, various studies have been performed to introduce planned treatment to an accurate target.^11^, ^12^, ^13^, ^14^ Setup verification for radiotherapy with an EPID has been studied by many authors.^15^, ^16^, ^17^ Uncertainties and variations exist in the positioning of SRS and stereotactic radiotherapy treatment techniques with non‐coplanar beams.^18^ The uncertainty in patient positioning with and without a surgical mask during thermoplastic immobilization has been studied.^19^ EPID dosimetry can be used to compare the delivered fluence with planned fluence using advanced radiotherapy techniques. Various studies have shown that the accuracy of the measured fluence is equivalent to that of other measurements.^20^, ^21^ The EPID can be used to verify the rotational accuracy of the gantry and multi‐leaf collimator (MLC) accuracy.^22^ The accuracy of the gantry position when acquiring EPID images also plays an important role. During patient isocenter verification, EPID images acquired with different gantry angles result in an incorrect isocenter shift, and thus incorrect treatment. The recording and verification system accepts deviations in the gantry angle of up to 2° when acquiring images with the EPID. If the EPID images are acquired at different gantry angles within 2°, it will result in an incorrect shift of the isocenter, and thus, incorrect treatment.

This study analyzed the effect of isocenter position accuracy at different gantry angles during digitally reconstructed reference radiograph (DRR) acquisition for portal image matching. This study also focused on the dosimetric effects of different gantry angle positions for isocenter verification and different isocenter positional errors during setup verification. This study is significant when CBCT or kV imaging is not available because of various factors and when techniques such as IMRT and 3DCRT are to be implemented using portal imaging.

## 2 METHODS AND MATERIALS

### 2.1 Patients selection and simulation

Thirty patients with head and neck cancer (carcinoma of the tongue, buccal mucosa, oropharynx, and pharynx) treated using IMRT at our center were prospectively included in this study between June 2022 and September 2022. Immobilization devices were created for all patients using a head and neck five‐pointer pushpin thermoplastic mask (CIVCO Medical Solutions, USA). Computed tomography (CT) was performed using a General Electric (GE) Healthcare CT. CT simulation was performed with a slice thickness of 2.5 mm from the vertex to the T8 vertebra. The CT images were exported to the Monaco ^TM^ version 5.51.10 treatment planning system (TPS) (Elekta Medical Solution).

### 2.2 Contouring and treatment planning

Contouring of the target volume and organs at risk was performed by a trained radiation oncologist according to the Radiation Therapy Oncology Group protocol.^23^ Following contouring, IMRT treatment plans were generated for all patients using Monaco^TM^ TPS (version 5.51.10). A digitally reconstructed reference radiograph (DRR) from the anterior to posterior (AP to PA) was obtained for all patients using Monaco^TM^ software. A reference DRR was created for all patients at a gantry and collimator angle of zero degrees. The generated DRR images were exported to the iView GT electronic portal imaging software (Elekta Medical Solution, version release 3.4 b162‐SP2).

### 2.3 Patient setup and imaging

The exported 0° gantry angle reference DRR images were imported from the iView GT software for all patients. The patients were positioned at the isocenter of the Elekta Synergy Platform^TM^ treatment couch with their customized immobilization mask. Using the double exposure option, positional images of the patients were acquired using EPID and iViewGT software. Actual positional images (AP to PA) were acquired for all patients at gantry angles of 0°, 0.5°, 1.0°, 1.5°, and 2.0°. The actual position images at different gantry angles were matched with the DRR reference images. The isocenter shifts were measured at different gantry angles for each patient in the lateral and longitudinal directions. The average isocenter shifts for different gantry angles were calculated for all patients. Using the average isocenter shift, the average isocenter difference in the lateral and longitudinal directions was calculated for gantry angles of 0 °to 0.5°, 0 °to 1.0°, 0 °to 1.5°, and 0 °to 2.0 °.

### 2.4 Quality assurance

The IMRT quality assurance (QA) procedure was performed using the Omnipro IMRT Matrixx software system (IBA, Germany) with a 1020 air‐vented ionization chamber at a resolution of 0.76 cm. Reference IMRT QA plans were generated using the Monaco^TM^ (version 5.51.10) TPS for all treatment plans in the static gantry angular position. Using the IMRT QA plan, the reference fluence was generated at a gantry angle of 0° in the coronal plane for all patients. First, the IMatriXX devices were positioned in the isocenter, IMRT QA plans were delivered, and the fluence was measured for all patients in the 0° gantry position. Then, the IMatriXX device was moved in the lateral direction with the calculated average isocenter shift difference for 0° to 0.5° gantry angle, and the IMRT QA fluence was measured for all patients. Similarly, the IMatriXX device was moved in the lateral direction with the calculated average isocenter shift difference from 0° to 1.0°, 0° to 1.5°, and 0° to 2.0° gantry angles, and the IMRT QA fluence was measured for all patients.

The gamma analysis was performed using gamma criteria of 3%, 3 mm, 2%, and 3 mm. Five IMRT QA fluence values were measured for each patient, and all the measured fluence values were compared with the reference fluence. The QA pass rate was analyzed and tabulated for a gantry angle of zero degrees and different isocenter shifts based on the gantry angle difference. The threshold pass rates for gamma 3%, 3 mm, and 2% 3 mm analyses were higher than 95%.

### 2.5 Data analysis

Using the isocenter shifts measured at different gantry angles, the average isocenter shifts in the lateral and longitudinal directions were measured at different gantry angles. Using the average of the isocenter shifts, the average differences in the lateral and longitudinal directions were calculated for gantry angles ranging from 0° to 0.5°, 0° to 1°, 0° to 1.5°, and 0° to 2.0°. The average gamma‐pass rates (3%, 3 mm and 2%, 3 mm) were calculated for a 0° gantry angle (isocenter) and for different shifts according to the gantry angles. The average gamma analysis pass rate was calculated for 0, 1.0, 2.0, and 3.0 mm isocenter shifts.

Statistical analyses were performed using SPSS software. To verify the systemic difference between the different gantry angles, statistical analysis was performed using one‐way repeated‐measures ANOVA for the isocenter shift between gantry angles of 0° to 0.5°, 0° to 1.0°, 0° to 1.5°, and 0° to 2.0°.^24^, ^25^ For pairwise comparisons, the Bonferroni method was used to analyze the *P* Values. Statistical significance was set at *P* < 0.05. Additionally, Mauchly's test was used to assess the sphericity. When the sphericity assumption was significant, the Greenhouse‐Geisser F value was used; otherwise, the corresponding sphericity assumption values were used. The Shapiro‐Wilk test was performed to check for normality. Similarly, the same statistical analysis test was performed to check the difference in isocenter shift with the actual isocenter using gamma analysis between 0 to 0.7 mm, 0 to 1.3 mm, 0 to 1.9 mm, and 0 to 2.5 mm.

## 3 RESULTS

**FIGURE 1:**
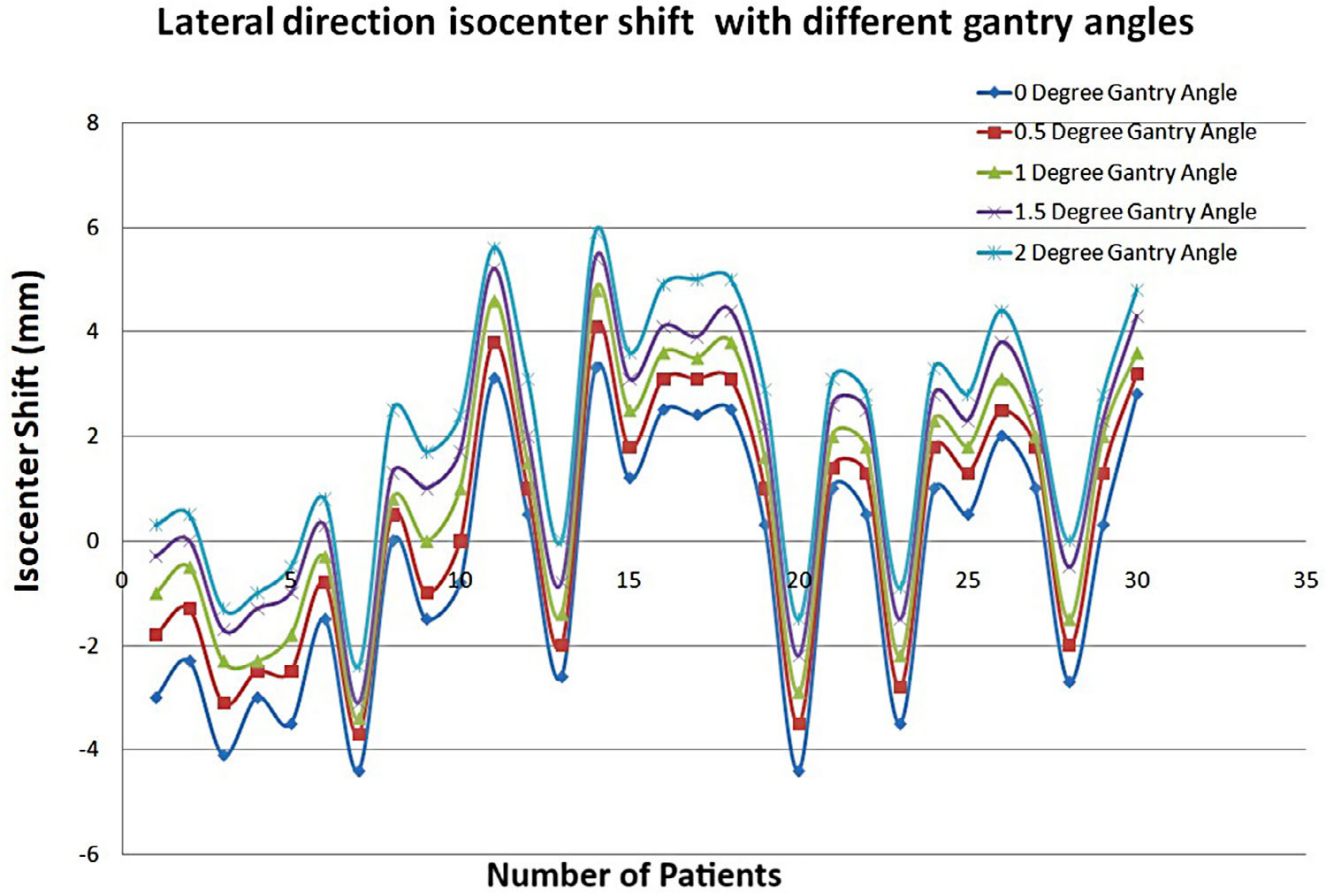
The isocenter shift for the lateral direction with different gantry angles.

**FIGURE 2:**
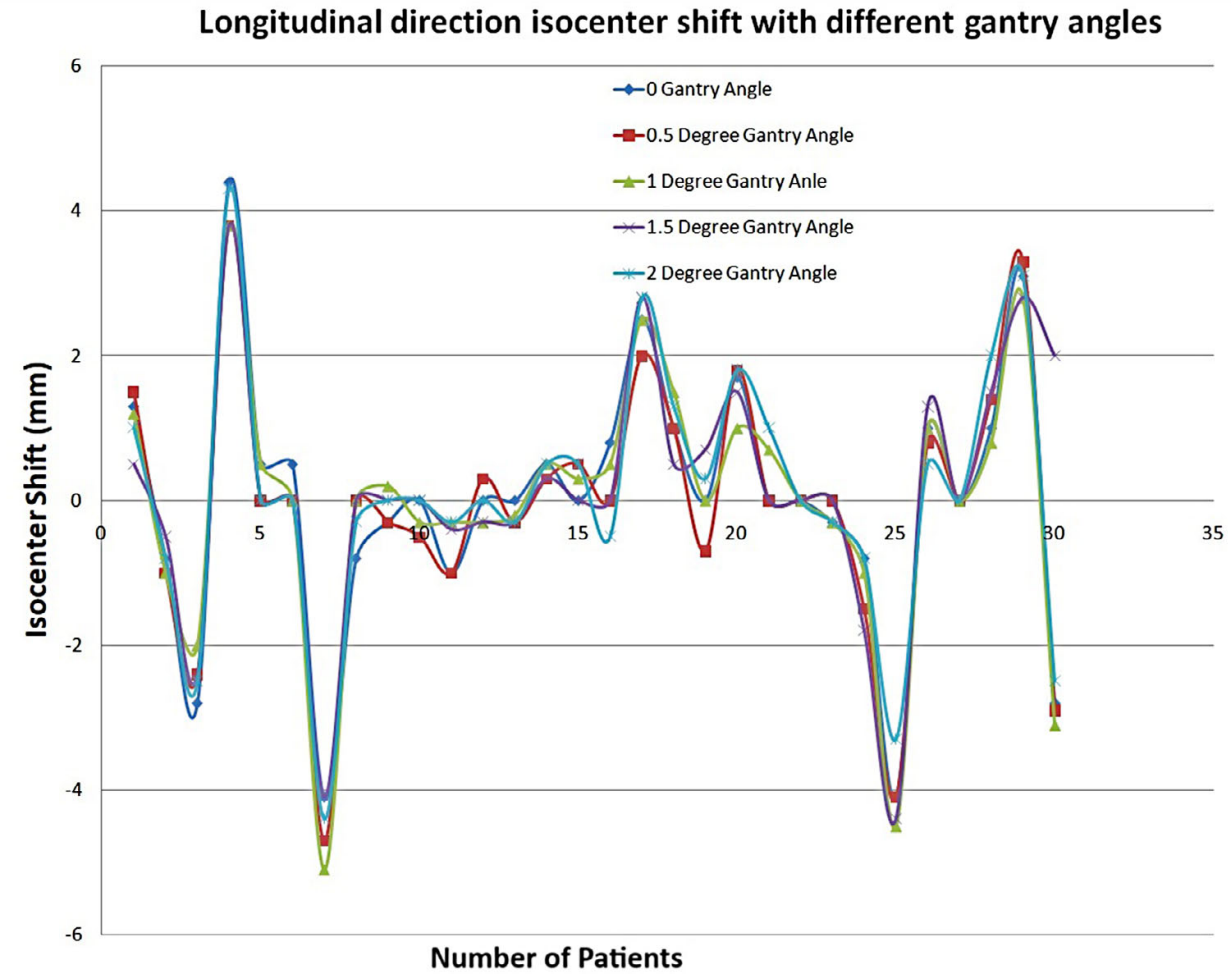
The isocenter shift for the longitudinal direction with the different gantry angles.

Figure 1 shows the isocenter shift in the lateral direction at different gantry angles. Table 1 lists the mean average positional errors of the isocenter in the lateral and longitudinal directions at different gantry angles. The average shift measured at different gantry angles of 0°, 0.5°, 1.0°, 1.5°, and 2.0° were (2.08 ± 0.238) mm, (2.79 ± 0.250) mm, (3.38 ± 0.256) mm, (4.00 ± 0.264) mm, and (4.60 ± 0.257) mm for lateral isocenter displacement, respectively. Figure 2 shows the isocenter shift in the longitudinal direction for different gantry angles. For the longitudinal direction, the average shift measured at different gantry angles of 0°, 0.5°, 1.0°, 1.5°, and 2.0° was (1.20 ± 0.247) mm, (1.23 ± 0.234) mm, (1.27 ± 0.234) mm, (1.20 ± 0.249) mm, and (1.25 ± 0.236) mm, respectively.

**TABLE 1:** Mean average isocenter positional errors for the lateral and longitudinal directions of the different gantry angles.

		Lateral direction		Longitudinal direction	
S.No	Gantry angle (°)	Mean Average isocenter positional error ± Standard deviation (mm)	95% Confidence Interval Lower Bound‐Upper Bond (mm)	Mean Average isocenter positional error ± Standard deviation (mm)	95% Confidence Interval Lower Bound‐ Upper Bond (mm)
1	0°	2.08 ± 0.238	1.59‐2.56	1.20 ± 0.247	0.70‐1.71
2	0.5°	2.79 ± 0.250	2.28‐3.31	1.23 ± 0.234	0.75‐1.71
3	1.0°	3.38 ± 0.256	2.85‐3.90	1.27 ± 0.234	0.79‐1.75
4	1.5°	4.00 ± 0.264	3.46‐4.54	1.20 ± 0.249	0.69‐1.71
5	2.0°	4.60 ± 0.257	4.08‐5.13	1.25 ± 0.236	0.77‐1.73

Table 2 shows the mean average isocenter positional error for different gantry angles with respect to the 0° gantry angle positional error for the lateral and longitudinal directions. The average difference was compared with the zero‐gantry angle. The average isocenter difference was 0.7 mm, 1.3 mm, 1.9 mm, and 2.5 mm for the lateral direction isocenter shift with respect to gantry angles of 0.5°, 1.0°, 1.5°, and 2.0°, respectively. The average isocenter difference was 0.02 mm, 0.07 mm, ‐0.00 mm, and ‐0.05 mm for isocenter shifts in the longitudinal direction with respect to gantry angles of 0.5°, 1.0°, 1.5°, and 2.0°, respectively. The percent deviations of the isocenter shift in the lateral direction were 25.63%, 38.50%, 48.08%, and 54.88% for gantry angles of 0.5°, 1.0°, 1.5°, and 2.0°, respectively. The percent deviations of the isocenter shift in the longitudinal direction were 1.95%, 5.49%, 0, and 3.76% for gantry angles of 0.5°, 1.0°, 1.5°, and 2.0°, respectively.

**TABLE 2:** Mean average isocenter positional error for different gantry angles with respect to the 0° gantry angle positional error for the lateral and longitudinal directions.

				Gantry angles 0° ‐ 0.5°			Gantry angles 0° ‐ 1.0°			Gantry angles 0° ‐ 1.5°			Gantry angles 0° ‐ 2.0°		
S.No	Positional error direction	ANOVA *P* value	*F* value	Mean Average isocenter positional difference from the 0° gantry angle (mm)	*P* Value	Percentage of deviation from the 0° gantry angle (%)	Mean Average isocenter positional difference from the 0° gantry angle (mm)	*P* Value	Percentage of deviation from the 0° gantry angle (%)	Mean Average isocenter positional difference from the 0° gantry angle (mm)	*P* Value	Percentage of deviation from the 0° gantry angle (%)	Mean Average isocenter positional difference from the 0° gantry angle (mm)	*P* Value	Percentage of deviation from the 0° gantry angle (%)
1	Lateral	<0.001^*^	742.55	0.72	<0.001^*^	25.63	1.30	<0.001^*^	38.50	1.92	<0.001^*^	48.08	2.53	<0.001^*^	54.88
2	Longitudinal	0.898	0.267	0.02	1.000	1.95	0.07	1.000	5.49	0.00	1.000	0	0.05	1.000	3.76

Repeated‐measures statistical analysis (ANOVA) revealed a significant *P* value (*P* < 0.001) for the isocenter shift in the lateral direction at different gantry angles. In the longitudinal direction, the statistical analysis did not reveal a significant *P* value for the isocenter shift at different gantry angles. Table 3 lists the mean average gamma‐pass percentage and standard deviation for different isocenter positional errors relative to the actual isocenter. The average lateral isocenter shift difference was applied to verify the gamma pass rate for different gantry angle errors. The gamma pass rate with gamma criteria (3%, 3 mm) was (96.62 ± 0.170)%, (94.43 ± 0.207)%, (92.29 ± 0.261)%, (89.90 ± 0.392)% and (87.06 ± 0.481)% with respect to 0 mm, 0.7 mm, 1.3 mm, 1.9 mm and 2.5 mm isocenter positional errors, respectively. The gamma pass rate with gamma criteria (2%, 3 mm) was (95.65 ± 0.314)%, (93.25 ± 0.482)%, (90.91 ± 0.608)%, (88.28 ± 0.717)% and (85.28 ± 0.799)% with respect to 0 mm, 0.7 mm, 1.3 mm, 1.9 mm and 2.5 mm isocenter positional errors, respectively.

**TABLE 3:** Mean average gamma‐pass percentage and standard deviation for different isocenter positional errors relative to the actual isocenter.

		(Gamma 3%, 3mm)		(Gamma 2%, 3mm)	
S.No	Isocenter Positional Error (mm)	Mean Average Gamma Pass percentage ± Standard deviation (%)	95% Confidence Interval Lower Bound ‐ Upper Bond (%)	Mean Average Gamma Pass percentage ± Standard deviation (%)	95% Confidence Interval Lower Bound ‐ Upper Bond (%)
1	0	96.62 ± 0.170	96.27‐96.97	95.65 ± 0.314	95.01‐96.29
2	0.7	94.43 ± 0.207	94.00‐94.85	93.25 ± 0.482	92.26‐94.24
3	1.3	92.29 ± 0.261	91.759‐92.82	90.91 ± 0.608	89.66‐92.15
4	1.9	89.90 ± 0.392	89.101‐90.70	88.28 ± 0.717	86.81‐89.74
5	2.5	87.06 ± 0.481	86.078‐88.04	85.28 ± 0.799	83.64‐86.91

Table 4 lists the mean average gamma pass percentage difference from the actual isocenter for different isocenter positioning errors, *P*‐values, and percentages of deviation from the actual isocenter. The gamma (3%, 3 mm) mean average gamma pass percentage from the actual isocenter position was (2.19 ± 0.154)%, (4.33 ± 0.236)%, (6.72 ± 0.384)%, and (9.56 ± 0.481)% corresponding to positional errors of 0–0.7 mm, 0–1.3 mm, 0–1.9 mm, and 0–2.5 mm, respectively. The gamma (2%, 3 mm) mean average gamma pass percentage from the actual isocenter position was (2.40 ± 0.284)%, (4.74 ± 0.426)%, (7.37 ± 0.545)%, and

**TABLE 4:** Mean average gamma pass percentage difference from the actual isocenter for different isocenter positional errors, standard deviations, *P*‐values, and percentages of deviation from the actual isocenter

		(Gamma 3%, 3mm)				(Gamma 2%, 3mm)			
S.No	Isocenter Positional Error from the actual Isocenter (mm)	Mean average Gamma Pass percentage difference from the actual position ± Standard deviation (%)	*P* Value	Percentage of deviation from the actual isocenter (%)	95% Confidence Interval for Difference^b^ Lower Bound‐ Upper Bond (%)	Mean average Gamma Pass percentage difference from the actual position ± Standard deviation (%)	*P* Value	Percentage of deviation from the actual isocenter (%)	95% Confidence Interval for Difference^b^ Lower Bound ‐ Upper Bond (%)
1	0‐0.7	2.19 ± 0.154	<0.001^*^	2.32	1.73‐2.66	2.40 ± 0.284	<0.001^*^	2.57	1.53‐3.26
2	0‐1.3	4.33 ± 0.236	<0.001^*^	4.69	3.61‐5.05	4.74 ± 0.426	<0.001^*^	5.21	3.45‐6.03
3	0‐1.9	6.72 ± 0.384	<0.001^*^	9.95	5.55‐7.89	7.37 ± 0.545	<0.001^*^	8.35	5.71‐9.02
4	0‐2.5	9.56 ± 0.481	<0.001^*^	10.98	8.10‐11.02	10.37 ± 0.624	<0.001^*^	12.16	8.47‐12.27

* shows statically significant values.

(10.37 ± 0.624)% corresponding to positional errors of 0–0.7 mm, 0–1.3 mm, 0–1.9 mm, and 0–2.5 mm, respectively. The repeated‐measures statistical analysis test (ANOVA) revealed a significant *P* value (*P* < 0.001) for both gamma criteria (3%, 3 mm and 2%, 3 mm) and gamma‐pass rates with respect to different isocenter positioning errors. The average gamma pass percentage with gamma 3%, 3 mm was 96.63%, 93.49%, 89.51% and 84.62% with respect to isocenter errors 0, 1.0, 2.0 and 3.0 mm, respectively.

## 4 DISCUSSION

IMRT improves target coverage and reduces the normal organ dose in head and neck radiotherapy. The accurate positioning of a patient during treatment is a critical aspect of treatment. It is also important to ensure that the isocenter is verified regularly.^10^ To ensure the isocenter position, daily imaging is a better choice for the verification of patient position before treatment.^14^ Before using EPID for clinical treatment, specific QA must be performed according to recommendations of the AAPM Task Group 58.^3^ The accuracy of the gantry position during image acquisition with the EPID plays an important role in verifying the accuracy of the isocenter.

This study aimed to investigate the effects of different gantry angles during EPID image acquisition for isocenter verification using reference DRR images. Different gantry angles were selected to examine their effects on the isocenter position verification. Moreover, we investigated the effects of different isocenter positional errors from the treatment isocenter while delivering beams with an incorrect isocenter using IMRT QA. Reference DRR images were acquired at the zero‐gantry angular position. If the DRR reference images are carelessly matched with the images acquired at other gantry angles, this will result in incorrect positioning of the isocenter. It is possible that the isocenter may shift for reasons such as the mechanical rotation of the gantry, which may affect patient treatment. Figures 1 and 2 show the isocenter shifts in the lateral and longitudinal directions for different gantry angles. Figure 1 clearly shows that the isocenter shift in the lateral direction always increased when the gantry angle increased and increased in the same direction. Figure 2 shows that the isocenter shift in the longitudinal direction did not change significantly when the gantry angle increased.

The results of the statistical analysis also show that the isocenter shift in the lateral direction changed significantly as the gantry angle increased. The average isocenter difference in the lateral direction increased with the gantry angle. The average isocenter difference in the longitudinal direction was approximately the same for all gantry angles. The percentage deviation in the lateral direction increased as the gantry angle increased from zero. There was no significant difference in the percentage of deviation in the longitudinal direction when the gantry angle was increased from the zero‐gantry angle. Patient positioning errors can occur when treatment is performed in a non‐co‐planer owing to the rotation of the couch.^18^ Once the systemic error is reduced, the margin of the PTV can be reduced to spare normal structures around the target volume.^12^

The IMRT QA results showed that when the position of the isocenter moved away from the actual isocenter, the gamma index (gamma 3%, 3 mm and gamma 2%, 3 mm) pass percentages decreased accordingly. The gamma‐pass percentage for the gamma criteria (2%, 3 mm) was less than 95%, because a lower detector resolution was used for the measurement.^26^ When the patient's position was not correctly reproduced, the setup errors were larger.^16^ The dose distribution in a highly conformal radiotherapy treatment plan changes if the patient's movement changes slightly or if isocenter positioning is performed incorrectly during treatment.^7^, ^8^ If the position of the isocenter differs from that of the actual isocenter by 1.0 mm, a significant treatment error occurs. To avoid these errors, the radiation therapist should ensure that the gantry angle matches the reference DRR image every time the EPID image is acquired. Therefore, it is necessary to perform routine QA of the gantry angle to avoid errors in positioning the isocenter during EPID imaging.^27^, ^28^ Several treatment centers use EPID for the following purposes: pretreatment patient position verification, treatment dose verification, MLC position verification, and in vivo dosimetry.^5^, ^22^ The EPID and IMatriXX were more accurate for patient‐specific QA before starting IMRT treatment.^21^ In centers where 3D imaging is not available for setup verification, the accuracy of 2D imaging is important for an accurate isocenter shift to avoid target misses. CBCT image verification improves the results, but results in an additional radiation dose to the patients and increased time for image review.^16^ In electron treatment, patient position verification using an EPID also plays an important role.^2^

In this study, we investigated only the lateral and longitudinal isocenter positional errors using images from the AP‐to‐PA EPID. The vertical isocenter position error was calculated similarly to the lateral isocenter position error based on the x‐, y‐, and z‐coordinates in isocenter verification.^18^

## 5 CONCLUSION

The results of this study showed that as the gantry angle increased, the average isocenter shift and percentage deviation with respect to the lateral direction also increased. The average isocenter difference showed that up to 0.5°, the isocenter shift was less than 1 mm. In the longitudinal direction, the isocenter shift showed a minimal effect compared with a gantry angle of zero. The results from IMRT QA showed that the percentage of gamma index deviation decreased as the isocenter position moved away from the actual isocenter. The dose distribution in a highly conformal radiotherapy treatment plan will change if the patient moves slightly or if the isocenter is positioned incorrectly during treatment. To avoid these errors, the radiotherapist should ensure that the gantry angle is matched with the reference DRR image at each acquisition of EPID imaging, and it is also necessary to perform routine quality assurance for the gantry angle to avoid isocenter position error during EPID imaging. From this study, we conclude that the acceptable deviation of the gantry angle during EPID imaging is less than 0.5°.

## CONFLICT OF INTEREST STATEMENT

The authors declare that they have no competing interests.

## ETHICS STATEMENT

Not applicable.
